# Supplementary material for: Evaluating and Enhancing the Preparation of Patients and Families before Pediatric Surgery
Source: Children (Basel). 2020 Aug 5;7(8):90. doi: 10.3390/children7080090 (PMC7464428; doi:10.3390/children7080090)
Supplement: Supplementary file 1 [file children-07-00090-s001.pdf]

Surgery 101 Survey Questions for Parent to complete

Please fill out the following questions in relation to your child's surgical procedure at the Alberta Children's Hospital. Completion of each question is optional.

*If your child has been to ACH for more than one surgery, please base your responses on your most recent experience.

By completing the below survey you are consenting to this data being used for research purposes through REB18-0272. Hard copies of consent forms are available upon request, please contact Karen Stetar - karen.stetar@albertahealthservices.ca for further information. Principal Investigator: Dr. Kerryn Carter.

Demographics:

1. What was your child's age at the time of surgery?
    - Dropdown list of ages 0-18

2. What is your child's sex?
    - Male
    - Female
    - Other

3. Where did you travel from to come to ACH?
    - Within Calgary
    - Less than 100 km (approx. 1 hour)
    - 100-250 km (approx. 1-2.5 hours)
    - Greater than 250 km

4. Has your child been diagnosed with an anxiety disorder?
    - Yes: Please specify _________
    - No

5. Has your child been diagnosed with any other behavioural or mental health disorder?
    - Yes: Please specify _________
    - No

6. Has your child been diagnosed with Autism Spectrum Disorder?
Note: Research has shown that children with ASD have increased anxiety surrounding perioperative interventions.
    - Yes
    - No

Other Medical conditions? ________________________

Details surrounding your child's surgery:

7. How long ago was your child's surgery?
    - Within the last month
    - 1-6 months ago
    - 6-12 months ago

- More than 1 year ago

8. How much in advance did you know about your child’s surgery?
- It was done urgently in hospital
- Within one week
- Within one month
- More than one month

  Do you want to add any further details about the context of the surgery? ___________________________

9. Did your child stay in the hospital after their surgery?
- No
- Yes, in the Day Surgery unit
- Yes, in a different unit (please specify) ____________________

10. What type of surgical procedure did your child have?
  - Urology
  - Neurosurgery
  - ENT
  - Ophthalmology
  - Plastics
  - General
  - Orthopedics
  - Interventional Radiology
  - Dental Surgery
  - Oral Maxillofacial surgery
  - Gastroenterology
  - Other: Please specify __________

Preparation for Surgery:

11. Is your child scared of going to the doctor/dentist?
    Never / Sometimes / Often / Always

12. Did your child understand that they were going for a surgical procedure?
    Not applicable / No / Somewhat / Yes

13. How anxious/nervous was YOUR CHILD before their surgery? (on a scale of 1-10)
    1 (not nervous at all) 10 (very nervous)

    Comments: textbox

14. How anxious/nervous were YOU before your child’s surgery? (on a scale of 1-10)
    1 (not nervous at all) 10 (very nervous)

    Comments: textbox

15. Did somebody provide you with information and/or resources to help you prepare for your child’s surgery? If so, who?

- Nurse
- Surgeon
- Family doctor
- Child Life Specialist
- Not applicable / urgent procedure
- Other: Please specify ____________________

16. Did you visit the Preoperative Anesthesia Clinic prior to your child's surgery?
    Yes
    No
    Can't remember

17. Overall, did you feel well prepared for your child's surgery? (on a scale of 1-10)
    1 (not well prepared) 10 (Well prepared)

18. Through what form of communication would you find it easiest to prepare for your child's surgery?
- Paper handouts
- Website
- App

19. Would you be likely to download an app that provided information and resources to help prepare for surgery? (on a scale of 1-10)
    1 (Not at all likely) 10 (Extremely likely)

20. Overall, how was your child's experience? (on a scale of 1-10)
    1 (very stressful) 10 (not stressful at all)

21. Is there any specific information you wish you had access to now that the surgery is completed?
- Yes
- No

    Please explain? ____________

22. Is there any further information you wish to share regarding you and your child's experience at ACH? Please let us know in the space provided below or feel free to contact us to discuss further at 403-955-7810.
    [Textbox to fill out response]

Surgery 101:

23. Did you participate in the Surgery 101 program?
Note: Surgery 101 is 2 hour program provided by Child Life Specialists at ACH to educate and prepare you and your child for their surgery, and includes viewing equipment similar to what will be used in surgery.

- No, I did not know about the Surgery 101 program. I likely would not have attended had I known.

- No, I did not know about the Surgery 101 program. I would have wanted to attend if I had known.
- No, I wanted to attend but my schedule couldn't accommodate it.
- No, I wanted to attend but distance from hospital couldn't accommodate it.
- No, I chose not to attend this program.
- Yes, I attended Surgery 101.

If you answered YES to question 23 please answer questions 24-29 If you answered no please proceed to question 30.

24. Where did you hear about the Surgery 101 program?
    - Nurse
    - Surgeon
    - Family doctor
    - Child Life Specialist
    - I found it on the website
    - From a friend or family member
    - Other, Please specify: _______________

25. Did you have to take time off from work to attend the Surgery 101 program?
    - Yes
    - No

26. Did you feel more prepared for your child's surgery after attending Surgery 101? (on a scale of 1-10)

    1 (not at all) 10 (much better prepared)

27. Did your child feel/seem more comfortable/prepared after attending the Surgery 101 program? (on a scale of 1-10)

    1 (not at all) 10 (much better prepared)

28. What was something that you learned from the Surgery 101 program?

    Textbox to fill out own response

29. What was something you wish you learned from the Surgery 101 program?

    Textbox to fill out own response

Optional Anxiety Assessment:

30. Pre-operative anxiety assessment (scoring based on validated STATE), additional 10-20 questions.

Thank you for participating in our survey.
